# Toxic epidermal necrolysis-like acute cutaneous lupus erythematosus: two cases report

**DOI:** 10.11604/pamj.2021.38.236.27303

**Published:** 2021-03-04

**Authors:** Asmae Abdelmouttalib, Mariame Meziane, Karima Senouci

**Affiliations:** 1 Dermatology and Venereology Department, Mohammed V University, Rabat, Morocco

**Keywords:** Acute cutaneous lupus erythematosus, toxic epidermal necrolysis, TEN-like ACLE, case report

## Abstract

Toxic epidermal necrolysis-like acute cutaneous lupus erythematosus (TEN-like ACLE) is a rare manifestation of systemic lupus erythematosus (SLE). Because of its rarity, little is known about this entity. In this report, we describe a case of two women previously diagnosed with SLE that presented TEN-like skin lesions. The common elements in both patients were the initial disposition of the lesions on the photoexposed areas, the positivity of Nikolsky´s sign, the discrete mucosal attrition compared to that observed during TEN, and the simultaneous appearance of dermatological lesions with an extra-cutaneous flare of lupus disease. The skin biopsy in both cases showed epidermal necrosis with an identification of lupus band on direct immunofluorescence. Systemic corticosteroids were used with a good evolution after 2 weeks. Skin damage is an indicator of disease activity, and careful search for extracutaneous involvement is obligatory to prevent further complications.

## Introduction

Toxic epidermal necrolysis-like acute cutaneous lupus erythematosus (TEN-like ACLE) is a rare manifestation of systemic lupus erythematosus (SLE) that mimics TEN clinically by the presence of widespread denudation and blistering; and histologically because it leads to keratinocyte apoptosis [1]. It is considered as an acute syndrome of apoptotic pan-epidermolysis (ASAP) which is a rare syndrome characterized by acute and massive cleavage of the epidermis resulting from hyperacute epidermal basal cell apoptotic injury [2]. The proposed mechanisms of the massive apoptotic injury shared by both TEN-like ACLE (which is an ASAP) and TEN include upregulation of Fas-Fas ligand interaction and the role for cytotoxic and/or autoimmune T cells, with cytokine release and subsequent amplification of the inflammatory cascade [3]. However, there are other distinguishing factors concerning the pathogenesis of SLE and TEN; in SLE, the key element is the ultraviolet light that increases the levels of chemokine CCL27 and induces keratinocyte apoptosis [4]. Moreover, TEN is associated with specific haplotypes affecting drug metabolism [5] and high levels of granulysin, which is the most critical cytotoxic molecule in TEN-induced apoptosis [6]. We report two cases with SLE who had presented TEN-like ACLE.

## Patient and observation

### First case

In October 2018, a 27-year-old woman presented with a 2-week history of a pruritic rash on her face and ears. Her past medical history was significant for SLE diagnosed 5 years ago with cutaneous, joint, and renal tropism. She was treated with different treatments; first, she received 6 Cyclophosphamide boluses with relay by Azathioprine for 3 years, stopped for hematological toxicity, then switched to Mycophenolate Mofetil which was stopped by the patient herself for 1 year due to lack of means. She also had a notion of septic arthritis of the knee having recurred despite well-adapted antibiotic treatment and her aunt was being treated for pulmonary tuberculosis. Therefore, she was hospitalized this time to carry out the SLE systematization assessment and to update her treatment. In the initial physical examination, we found a malar rash; erythematous plaques on her face, ears, and upper limbs; and a diffuse non-scarring alopecia. She also had arthralgia especially in her left knee, with swelling in that area.

**Figure 1:**
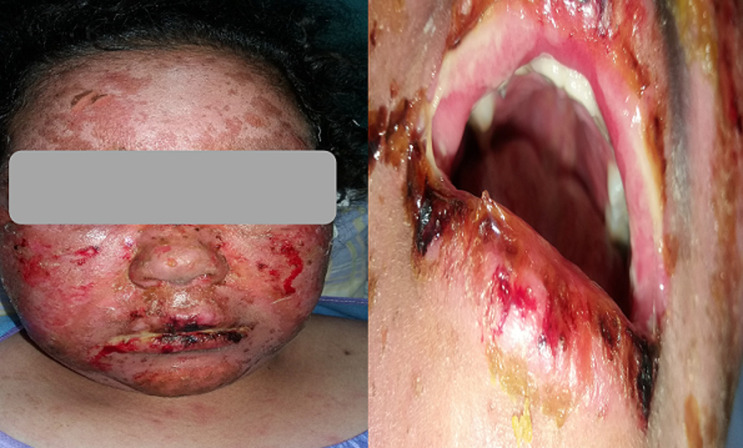
first case with erythema and post bullous erosions in the face with crusty cheilitis

**Figure 2:**
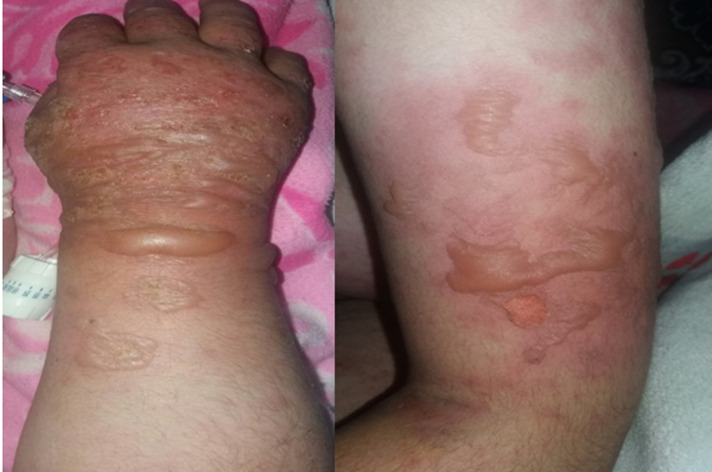
first case with erythema and bubbles in arms and back of the hand

A small left knee effusion was aspirated and the culture was positive for *Mycobacterium tuberculosis*; therefore, anti-tuberculous therapy (Rifampicin, Isoniazid, Pyrazinamide, and Ethambutol) was initiated. The cutaneous symptomatology worsened after 15 days from the start of tuberculosis treatment with extended rash at the trunk and neckline, erythematous and edematous plaques, and blisters in the photo-exposed areas. Nikolsky's sign was positive, the affected skin surface was estimated to be 30%, and the mucosal involvement was limited to a simple cheilitis (Figure 1, Figure 2). The antituberculosis treatment was initially discontinued because we had thought of the TEN.

Laboratory abnormalities showed proteinuria elevated to 2g/24h, bicytopenia (leucopenia at 3500 per cubic milliliter and anemia with hemoglobin at 10 grams per dl), an elevated anti-double stranded deoxyribonucleic acid (DNA) antibody with negative anti-histone antibodies and consumption of the complement C3 and C4. Moreover, skin biopsy showed diffuse epidermal necrosis, sub-epidermal detachment, interface dermatitis and presence of mucinosis. On direct immunofluorescence (DIF), a deposition of IgM and IgG in the basement membrane (lupus band) was present. Given the clinical history, lesion morphology, pathologic finding and laboratory studies, the diagnosis of TEN-like ACLE was established. After the administration of systemic corticosteroids, we have observed a healing of the skin lesions after two weeks. Anti-tuberculous treatment was reintroduced without any particular incident and Cyclophosphamide boluses were subsequently started. Joint swelling resolved within two months after the initiation of anti-tuberculous treatment and full joint range of motion was restored. There has been no recurrence of the bullous eruption to date.

### Second case

**Figure 3:**
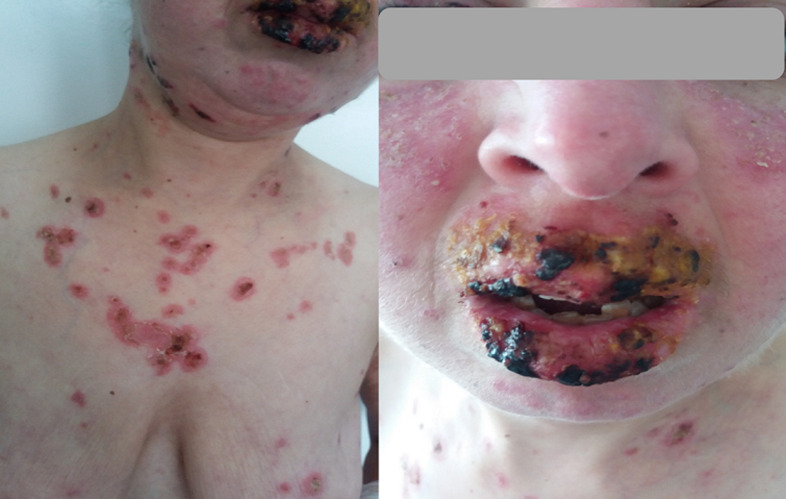
second case with erythematous and squamous plaques on the face and chest with crusty cheilitis

A 30-year-old woman presented to the emergency department for a pruritic rash that began on her face with rapid extension to her back, chest, and arms in three days. In her past medical history, we noted a SLE diagnosed the year before with malar rash, arthralgia, and lupus nephropathy confirmed based on renal biopsy puncture. She was receiving Hydroxychloroquine, low dose prednisone and Captopril (converting enzyme inhibitor) with Cyclophosphamide boluses (she was on her 3^rd^ bolus that she had received 15 days ago). A new medication was not reported. The Mucocutaneous examination showed erythematous and squamous plaques on malar cheeks, arms and chest; vesicles and blisters on the cleavage and a crusty cheilitis without lesions on the oral cavity (Figure 3). A morbilliform eruption on the sun-exposed areas of the upper trunk and arms was also described. The Nickolsky´s sign was positive, the affected skin surface was estimated to 30% and the patient was afebrile with normal vital signs.

**Figure 4:**
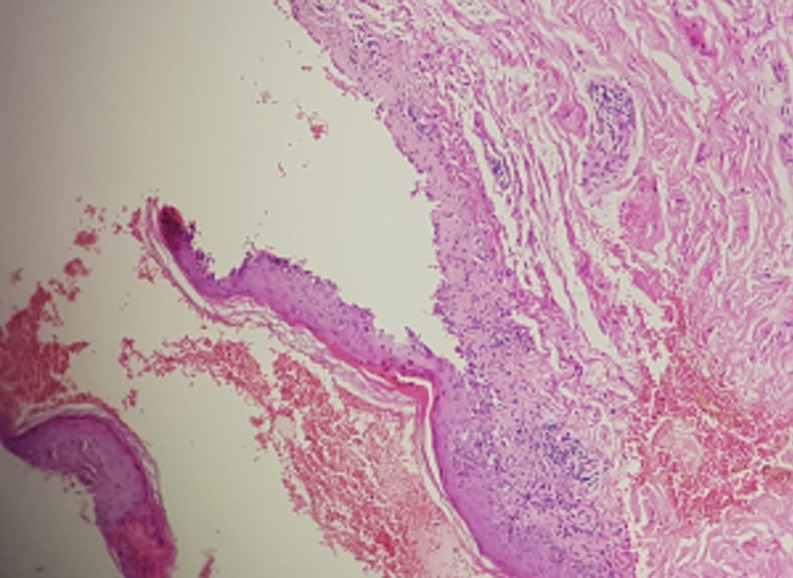
second case: histological section showing epidermal necrosis and interface dermatitis

Laboratory abnormalities included the following: an elevated anti-double stranded DNA antibody, negative anti-histone antibodies, consumption of the complement C3 and C4, middle leukopenia (white blood cell count of 4000 per cubic milliliter) and elevated proteinuria. A skin biopsy was taken from the chest; microscopic examination showed a superficial and deep perivascular infiltrate of lymphocytes and a sub epidermal bulla (Figure 4); the hair follicles were atrophic and eaten away by lymphoid elements and mucin deposits identified by the alcian blue coloring. Direct immunofluorescence demonstrated granular IgM and C3 along the basement membrane zone. The diagnosis of TEN-like ACLE has been confirmed, and the patient was treated by intravenous methylprednisolone for five days, followed by oral prednisone that was slowly tapered with complete healing of the lesions within 2 weeks. The other treatments (Hydroxychloroquine and Captopril) were continued and she received her 3^rd^ Cyclophosphamide bolus in the following 15 days as planned. There has been no recurrence of this symptomatology to date.

## Discussion

Both cases we present illustrate the challenge in diagnosing TEN-like SLE in patients previously diagnosed with SLE especially in the presence of a recent use of drugs and therefore not to wrongly consider it as a toxidermy (TEN). In our patients, the diagnosis of TEN was considered initially as there was an acute onset of skin lesions and a positive Nikolsky's sign; moreover, in the first case, the notion of the recent use of anti-tuberculosis drugs and the 15-day delay of symptom onset were also factors in favor of this diagnosis. However, the diagnosis was corrected afterwards, and we retained the diagnosis of TEN-like ACLE due to the strict photo distribution of lesions, the discrete mucous membrane involvement, the absence of flu-like prodrome and the flare-up of SLE that was concomitant with skin manifestations. There was also the presence of the lupus band at the DIF associated with epidermal necrosis and presence of mucinosis. In addition, the negativity of the anti-histone antibodies helped to eliminate drug-induced lupus. Our cases also illustrates that the presentation of TEN-like SLE can mimic others bullous eruptions in SLE patients like bullous SLE, Rowell’s syndrome and TEN. The clinical and pathological features for of the main bullous eruptions during the SLE are well described in the literature (Table 1) [1, 2, 7, 8].

**Table 1:** differential diagnosis of the bullous eruption of our patients

Bullous eruption	Clinical findings	Pathologic findings	DIF
**TEN-like ACLE**	-Prior or acute diagnosis of lupus	-Full thickness epidermal necrosis often with marked dyskeratosis extending down adnexae,	Granular IgG, IgM, and/or C3 blinding at the DEJ
	-Vesicobullous lesions with photo- accentuated epidermal sloughing, body surface area >30%.		
	-Nickolsky’s sign can be present or not		
	-Patient may have a minimal mucosal involvement, particularly oral	-Interface and dermal inflammation and mucin	
**Bullous LE**	-Tense vesicobullous lesions on sun-exposed areas without generalization to the rest of the body	Sub epidermal blister with neutrophils but without epidermal necrosis	Linear or granular IgG, IgM, IgA and C3 visualized at the DEJ
	-Occasional mucus membrane involvement		
**TEN**	-Flu-like prodrome followed by dusky macules that coalesce	Full thickness epidermal necrosis with sparse to absent lymphocytic infiltrate	Negative
	-Subsequent development of bullae along with epidermal sloughing >30% body surface area		
	-Severe mucous membrane involvement.		
	-Most cases associated with a causative drug		
**Rowell’s syndrome**	*Major criteria:	Necrotic keratinocytes accompanied by sub epidermal blister and middle lymphocytic infiltrate	Negative
	-Erythema multiform-like lesions, presence of LE (discoid, systemic, or subacute), speckled pattern of antinuclear antibody		
	*Minor criteria:		
	-presence of anti RO body and/or anti-La antibodies, positive RF, chilblains lesions		


Abbreviations: %, percent; >, greater than; ACLE, acute cutaneous lupus erythematosus; C3, third component of complement; CLE, cutaneous lupus erythematosus; DEJ, dermoepidermal junction; DIF, direct immunofluorescence; IgA, immunoglobulin A; IgG, immunoglobulin G; IgM, immunoglobulin M; LE, lupus erythematosus; RF, rheumatoid factor; TEN, toxic epidermal necrolysis

In the literature, 43 cases of TEN-like ACLE were reported; 37 women and 6 men, and we have reported two additional cases. The ratio of women to men is 6.6/1 and the mean diagnosis age is 43 years [1]. The most of patients (60%) had a confirmed diagnosis of SLE or subacute cutaneous lupus erythematosus (SCLE) prior to developing TEN-like ACLE [1]. Initiation of a new medication had been reported in some cases of literature; the target drug, however, can be restarted later without an adverse cutaneous event [1]. According to a study [9], all patients who develop TEN-like ACLE had internal organ involvement (hematologic and renal) and a high Systemic Lupus Erythematosus Disease Activity Index score. The Treatment of TEN-like ACLE is mainly systemic corticosteroids either alone or combined with one or more additional therapies (Hydroxychloroquine, intravenous immunoglobulin, or Mycophenolate Mofetil) [1]. In terms of mortality, 89% of the TEN-like ACLE patients recovered from their TEN-like lesions while only 68% to 80% of patients with TEN survived [10]. For recurrences, none had disease recurrence [6].

## Conclusion

Our experience illustrates the interest to be able to diagnose TEN-like ACLE as a cause of bullous eruption even when there is a recent use of drugs because the incidence of TEN-like ACLE may be inflated because of misclassification of this entity as TEN. The diagnosis of TEN-like ACLE is often made retrospectively after correlation between different clinical, serologic, and histopathological data. Skin damage is an indicator of disease activity, and careful search for extracutaneous involvement to prevent further complications is obligatory. The knowledge of this entity allows an early diagnosis and consequently an adapted therapeutic management.
